# Mortalidade por Insuficiência Cardíaca durante a Pandemia da COVID-19: *Insights* de uma Coorte de Hospitais Públicos no Brasil

**DOI:** 10.36660/abc.20220080

**Published:** 2022-09-06

**Authors:** Miguel M. Fernandes-Silva, Eduardo Leal Adam, Sabrina Bernardez-Pereira, Suzana Alves Silva, Luiz Guilherme Passaglia, Kleber Renato Ponzi Pereira, Marco Antônio Vieira Guedes, João David de Souza, Ângelo Amato Vincenzo de Paola, Maria Alayde Mendonça Rivera, Elmiro Santos Resende, Denilson Campos de Albuquerque, Fernando Bacal, Antônio Luiz Pinho Ribeiro, Louise Morgan, Sidney C. Smith, Fábio Papa Taniguchi

**Affiliations:** 1 Universidade Federal do Paraná Curitiba PR Brasil Universidade Federal do Paraná , Curitiba , PR – Brasil; 2 Hospital do Coração São Paulo SP Brasil Hospital do Coração – HCor, São Paulo , SP – Brasil; 3 Universidade Federal de Minas Gerais Belo Horizonte MG Brasil Universidade Federal de Minas Gerais , Belo Horizonte , MG – Brasil; 4 Fundação Hospital de Clínicas Gaspar Vianna Belém PA Brasil Fundação Hospital de Clínicas Gaspar Vianna , Belém , PA – Brasil; 5 Hospital Ana Nery Salvador BA Brasil Hospital Ana Nery , Salvador , BA – Brasil; 6 Hospital de Messejana Fortaleza CE Brasil Hospital de Messejana – Dr. Carlos Alberto Studart Gomes, Fortaleza , CE – Brasil; 7 Universidade Federal de São Paulo São Paulo SP Brasil Universidade Federal de São Paulo , São Paulo , SP – Brasil; 8 Santa Casa de Misericórdia de Maceió Maceió AL Brasil Santa Casa de Misericórdia de Maceió , Maceió , AL – Brasil; 9 Universidade Federal de Uberlândia Uberlândia MG Brasil Universidade Federal de Uberlândia , Uberlândia , MG – Brasil; 10 Universidade Estadual do Rio de Janeiro Rio de Janeiro RJ Brasil Universidade Estadual do Rio de Janeiro , Rio de Janeiro , RJ – Brasil; 11 Instituto do Coração São Paulo SP Brasil Instituto do Coração , InCor, São Paulo , SP – Brasil; 12 American Heart Association Inc Dallas Texas USA American Heart Association Inc , Dallas , Texas – USA; 13 University of North Carolina at Chapel Hill Chapel Hill North Carolina USA University of North Carolina at Chapel Hill , Chapel Hill , North Carolina – USA

**Keywords:** Doenças Cardiovasculares/complicações, Pandemia, COVID-19, Insuficiência Cardíaca, Hospitais Públicos, Mortalidade, Assistência à Saude/métodos

## Introdução

O Brasil é um dos principais países afetados pela pandemia causada pelo novo coronavírus (COVID-19), com mais de meio milhão de mortes em junho de 2021, o segundo maior número de mortes no mundo. ^1^ O surto de COVID-19 parece ter afetado a prestação de cuidado em saúde a pacientes sem COVID-19, particularmente pacientes com doenças cardiovasculares (DCV), aumentando o número de mortes pela pandemia. Estudos mostraram uma redução em hospitalizações e um aumento em mortalidade por DCV durante o surto de COVID-19 em muitos países. ^2 - 8^ Contudo, a maioria desses estudos envolveram pacientes com síndrome coronariana aguda (SCA) em países desenvolvidos, com poucos dados sobre admissões por insuficiência cardíaca (IC), e as causas para o aumento na mortalidade não foram bem esclarecidas. Nós avaliamos as mudanças na gravidade, causas de descompensação da IC, e mortalidade em 30 dias de pacientes admitidos por IC em hospitais públicos incluídos no programa Boas Práticas Clínicas (BPC) em Cardiologia no Brasil durante a pandemia da COVID-19 e comparadas com períodos prévios.

## Métodos

Realizamos um estudo do tipo coorte usando dados do programa BPC, o qual encontra-se em andamento, e consiste em uma adaptação da iniciativa *Get With The Guidelines* , para melhorar a qualidade do cuidado cardiovascular e desfechos dos pacientes no Brasil. O delineamento, racional e procedimentos do estudo foram previamente publicados. ^9^ Incluímos pacientes consecutivos, com idade igual ou superior a 18 anos, admitidos com IC descompensada em hospitais públicos terciários afiliados ao Sistema Único de Saúde (SUS) durante a pandemia por COVID-19 entre 12 de março de 2020 a 31 de outubro de 2020 (semanas epidemiológicas 11 a 44). Os pacientes foram comparados com pacientes admitidos nos mesmos hospitais durante as mesmas semanas epidemiológicas em 2019 e 2018.

Características basais específicas à IC foram coletadas prospectivamente durante a hospitalização, por investigadores locais treinados, usando prontuários médicos e entrevistas estruturadas com o paciente. Calculou-se o escore do algoritmo do registro ADHERE – *Acute Decompensated Heart Failure National Registry* (ADHERE) – usando uma abordagem do tipo *stepwise* de acordo com medidas de pressão arterial, creatinina, e nitrogênio ureico sanguíneo, para estratificar pacientes com IC em risco baixo, intermediário e alto. ^10 , 11^ O desfecho foi mortalidade por todas as causas em um seguimento de 30 dias. Óbito e data de óbito foram verificados por meio de prontuários médicos, descendentes e atestados de óbito.

Consentimento informado foi obtido de cada paciente, e o estudo foi aprovado pelo comitê de ética do centro coordenador do estudo (no. 48561715.5.1001.0060).

### Análise estatística

As variáveis contínuas foram avaliadas quanto à distribuição normal usando a forma, simetria e curtose da distribuição, e o teste de Kolmogorov-Smirnov se necessário. Nós comparamos características clínicas, medidas de gravidade e tratamento entre os períodos de tratamento usando o teste t não pareado para variáveis com distribuição normal, apresentadas como média ± desvio padrão, e o teste de Mann-Whitney para variáveis sem distribuição normal, apresentadas como mediana (percentis 25 e 75). Os dados categóricos foram apresentados como proporções e comparados usando o teste do qui-quadrado. Para avaliar a associação entre os períodos do estudo e os desfechos, nós calculamos estimativas de Kaplan-Meier e comparamos a probabilidade de mortalidade por todas as causas usando o teste de log-rank. Realizou-se análise de regressão (riscos proporcionais) de Cox ajustado por idade, sexo, etiologia da IC (isquêmica, doença de Chagas, outros), fração de ejeção do ventrículo esquerdo (FEVE), terapia de ressincronização cardíaca (TRC), cardioversor desfibrilador implantável (CDI), e nível educacional, e estratificado por renda, doença renal crônica (DRC) e IC prévia. A suposição de risco proporcional foi testada com base nos resíduos de Schoenfeld, sem violação da suposição de risco proporcional para os grupos estudados. Valores de p<0,05 foram considerados estatisticamente significativos. As análises foram realizadas usando o programa Stata versão 15.1 (Stata Corp., College Station, TX).

## Resultados

Durante as semanas epidemiológicas 11 a 44 nos anos 2018, 2019 e 2020, um total de 1084 pacientes foram admitidos com IC em sete centros elegíveis, representando seis estados (Alagoas, Bahia, Ceará, Minas Gerais, Pará e São Paulo) no Brasil. Desses pacientes, nós excluímos 218 por ausência de medidas de pressão arterial sistólica (n=86), creatinina (n=43), ureia no sangue (n=20) ou perda de seguimento (n=69). A análise final foi realizada em 866 pacientes admitidos por IC.

Em comparação aos dois anos anteriores, observamos uma redução de 20% no número de pacientes admitidos com IC durante a pandemia de COVID-19, principalmente nos primeiros quatro meses (9,2 ± 4,2 internações por semana durante a pandemia *vs* . 11,6 ± 3,0 antes da pandemia).

A maioria das características basais permaneceram as mesmas, mas a FEVE foi mais baixa. Doença de Chagas e doença isquêmica do coração foram as etiologias mais comuns de IC, e história de DRC foi mais prevalente durante a pandemia. Ainda, a gravidade da IC na admissão parece ter aumentado durante o surto da COVID-19. Embora os perfis clínicos não foram diferentes entre os períodos de estudo, os pacientes admitidos durante o surto da COVID-19 apresentaram escore de risco ADHERE significativamente pior ( Tabela 1 ).

**Tabela 1:** Características na admissão dos pacientes internados por insuficiência cardíaca antes e durante o surto da COVID-19

	Antes do surto da COVID-19 (2018 - 2019)	Durante o surto da COVID-19 (2020)	Valor p

	n=637	n=229	
Idade, anos	59,83 ± 16,00	61,00 ± 14,05	0,33
Mulheres, n(%)	275 (43,2)	93 (40,6)	0,50
IMC, Kg/m ^2^ *	26,16 ± 5,56	27,03 ± 6,30	0,05
Etiologia*			**0,028**
Isquêmica	109 (17,1)	47 (20,5)	
Chagas	54 (8,5)	31 (13,5)	
Outros	474 (74,4)	151 (65,9)	
FEVE, %	43,51 ± 17,62	37,33 ± 15,07	< 0,001
IC prévia, n(%)	395 (62,0)	153 (66,8)	0,20
DRC, n(%)	66 (10,4)	50 (21,8)	< 0,001
TRC, n(%)	3 (0,5)	3 (1,3)	0,19
CDI, n(%)	44 (6,9)	16 (7,0)	0,97
Baixo nível educacional, n (%)	265 (41,6)	119 (52,0)	0,007
Baixa renda, n (%)*	469 (73,7)	170 (74,6)	0,81
Tempo de internação, dias	19,0 [10,0, 33,0]	17,0 [9,0, 28,0]	0,17
**Perfil clínico**			**0,62**
Quente e seco	56 (10,3)	13 (7,7)	
Quente e úmido	368 (67,9)	114 (67,9)	
Frio e úmido	92 (17,0)	34 (20,2)	
Frio e seco	26 (4,8)	7 (4,2)	
**Risco ADHERE, n(%)**			**0,009**
Baixo	290 (45,5)	79 (34,5)	
Intermediário	319 (50,1)	134 (58,5)	
Alto	28 (4,4)	16 (7,0)	

IMC: índice de massa corporal; IC: insuficiência cardíaca; DRC: doença renal crônica; TRC: terapia de ressincronização cardíaca; CDI: cardioversor desfibrilador implantável; BUN: nitrogênio ureico no sangue. * Dados do perfil clínico de 156 pacientes; dados de índice de massa corporal de 56 pacientes; dados de renda de dois pacientes; e dados de fração de ejeção do ventrículo esquerdo de 23 pacientes eram faltantes.

**Figura 1:**
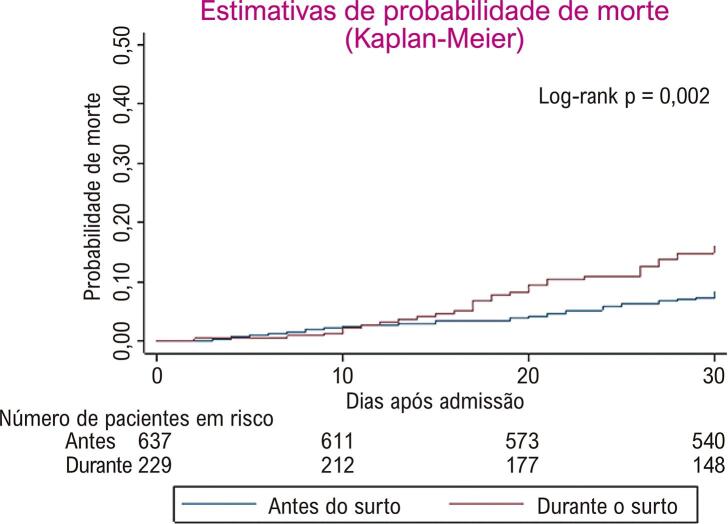
Estimativas de Kaplan-Meier da probabilidade de morte em pacientes admitidos por insuficiência cardíaca e antes e durante o surto de COVID-19. Antes do surto de COVID-19: semanas epidemiológicas 11 a 44 em 2018 e 2019; durante o surto de COVID-19: semanas epidemiológicas 11 a 44 em 2020

Mortalidade por todas as causas de pacientes internados por IC aumentou significativamente durante o surto da COVID-19 ( Figura 1 ). No período de acompanhamento de 30 dias, 50/637 (7,8%) e 31/229 (13,5%) pacientes morreram antes e durante o surto da COVID-19, respectivamente. Após ajuste quanto a potenciais fatores de confusão no basal, o risco de morte em 30 dias foi aproximadamente duas vezes maior em pacientes admitidos durante o surto da COVID-19 (HR ajustado = 1,89 [IC95% 1,19, 3,03]; *p* =0,007), em comparação aos dois anos anteriores.

**Figura 2:**
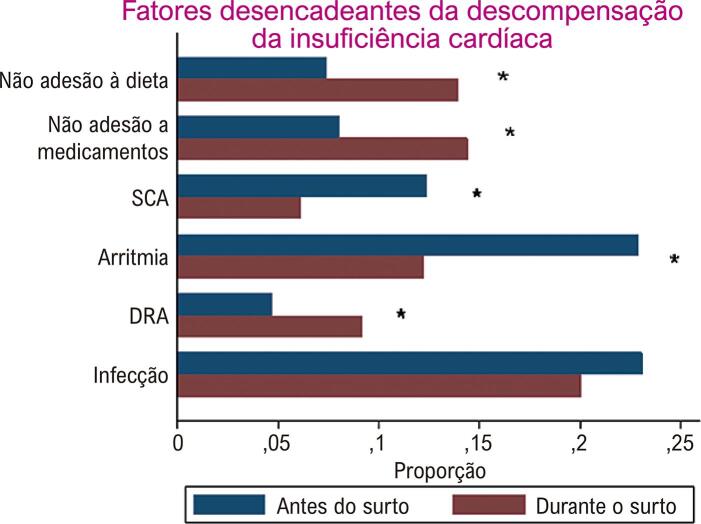
Fatores desencadeantes de descompensação da insuficiência cardíaca por período de estudo. SCA: síndrome coronariana aguda; DRA: doença renal aguda. * p<0,05 pelo teste do qui-quadrado comparando as proporções entre os dois períodos. Antes do surto de COVID-19: semanas epidemiológicas 11 a 44 em 2018 e 2019; durante o surto de COVID-19: semanas epidemiológicas 11 a 44 em 2020

Uma baixa adesão às recomendações de tratamento medicamentoso ou dietético, e doença renal aguda foram mais comumente relatados como fatores desencadeantes de descompensação de IC durante o surto de COVID-19, em comparação ao período anterior ( Figura 2 ). A frequência de infecção como razão de descompensação durante o surto da COVID-19 não foi diferente em comparação a antes do surto.

## Discussão

Neste grande registro de hospitalizações por IC no Brasil, nossos principais achados foram: 1) a mortalidade em 30 dias aumentou aproximadamente duas vezes durante o surto de COVID-19 em comparação a antes da pandemia; 2) pacientes com IC eram mais propensos a desenvolverem IC descompensada por baixa adesão ao tratamento e por doença renal aguda; e 3) os pacientes foram admitidos em condições piores, como indicado por um pior escore de risco ADHERE. Esses achados ajudam a entender o impacto da pandemia em pacientes com IC no Brasil, afetando o comportamento dos pacientes, interrompendo a prestação de serviços de saúde, e aumentando o risco de morte por IC aguda.

Até o momento, existem poucos relatados do impacto da pandemia da COVID-19 sobre pacientes com IC. Similar a nossos achados, estudos da Alemanha e da Inglaterra descreveram que a mortalidade em curto prazo aumentou significativamente entre pacientes admitidos por IC durante o início da pandemia, o que não foi descrito em estudos posteriores conduzidos na Dinamarca. ^2 , 4 , 5^ Tal fato indica que pacientes com DCV foram afetados em graus diferentes, dependendo do nível de resposta de cada país à pandemia. Nosso estudo mostrou um aumento na mortalidade por IC aguda em um país amplamente afetado, e que tal impacto teve duração mais longa que os primeiros meses da pandemia.

As causas para uma maior mortalidade são multifatoriais. A não adesão ao tratamento dietético e medicamentoso como fatores desencadeantes de IC descompensada durante a pandemia da COVID-19 pode fornecer *insights* sobre os mecanismos para esses piores desfechos. O tratamento da IC é complexo, e geralmente requer uma abordagem multidisciplinar centrado no paciente para melhorar a adesão ao tratamento em longo prazo. ^12^ Os piores desfechos da IC aguda pode refletir uma ruptura na prestação do cuidado no ambiente ambulatorial. Os serviços ambulatoriais foram interrompidos, e as equipes de cardiologia foram reorganizadas e transferidas para a prestação de cuidado da COVID-19 em muitos centros, alterando o foco das medidas efetivas essenciais para reduzir mortalidade dessa população. Além disso, a perda do apoio social devido ao isolamento social pode ter afetado a continuidade do tratamento, particularmente entre indivíduos vulneráveis.

Vale notar que as orientações para o manejo de DCV durante a pandemia da COVID-19 focaram principalmente SCA, mas pacientes com IC também foram muito afetados pela pandemia. Esforços são necessários para continuar a prover cuidado adequado a esses pacientes. ^13^ Políticas de saúde visando essa população, incluindo estratégias para se manter a prestação de cuidado no ambulatório, tais como telemedicina e monitoramento remoto, pode ajudar a reduzir a mortalidade durante a pandemia.

Nosso estudo tem limitações: incluímos somente hospitais públicos, a maioria hospitais universitários. Ainda, todos os hospitais em nosso estudo participavam do programa BPC, cujo objetivo é melhorar a qualidade do cuidado de DCV e desfechos dos pacientes, de modo que as internações no estudo podem não refletir as internações por IC em todos os hospitais públicos no Brasil. Finalmente, nossos resultados não representam os pacientes que não foram internados ou aqueles que não vieram ao hospital.

## Conclusão

Neste grande registro de pacientes admitidos com IC em hospitais públicos incluídos no programa BPC no Brasil, um dos países mais afetados pela pandemia da COVID-19, a baixa adesão ao tratamento e doença renal aguda foram as principais causas da descompensação da insuficiência cardíaca, e a mortalidade em 30 dias aumentaram duas vezes durante o surto da COVID-19 em comparação a períodos anteriores. Estratégias de saúde pública em resposta à pandemia deveria garantir manutenção do cuidado a pacientes com IC, particularmente nos países mais afetados.

## * Material suplementar

Para informação adicional, por favor,clique aqui.
