# Supplementary material for: Mortalidade por Insuficiência Cardíaca durante a Pandemia da COVID-19: Insights de uma Coorte de Hospitais Públicos no Brasil
Source: Arq Bras Cardiol. 2022 Sep 6;119(5):804–8. [Article in Portuguese] doi: 10.36660/abc.20220080 (PMC9750211; doi:10.36660/abc.20220080)
Supplement: Translation

# Translation: Heart Failure Mortality during COVID-19 Pandemic: Insights from a Cohort of Public Hospitals in Brazil

**Authors:** Miguel M. Fernandes-Silva, Eduardo Leal Adam, Sabrina Bernardez-Pereira, Suzana Alves Silva, Luiz Guilherme Passaglia, Kleber Renato Ponzi Pereira, Marco Antônio Vieira Guedes, João David de Souza, Ângelo Amato Vincenzo de Paola, Maria Alayde Mendonça Rivera, Elmiro Santos Resende, Denilson Campos de Albuquerque, Fernando Bacal, Antônio Luiz Pinho Ribeiro, Louise Morgan, Sidney C. Smith, Fábio Papa Taniguchi

**Affiliations:** 1 Brazil Universidade Federal do Paraná, Curitiba, PR – Brazil; 2 Brazil Hospital do Coração – HCor, São Paulo, SP – Brazil; 3 Brazil Universidade Federal de Minas Gerais, Belo Horizonte, MG – Brazil; 4 Brazil Fundação Hospital de Clínicas Gaspar Vianna, Belém, PA – Brazil; 5 Brazil Hospital Ana Nery, Salvador, BA – Brazil; 6 Brazil Hospital de Messejana – Dr. Carlos Alberto Studart Gomes, Fortaleza, CE – Brazil; 7 Brazil Universidade Federal de São Paulo, São Paulo, SP – Brazil; 8 Brazil Santa Casa de Misericórdia de Maceió, Maceió, AL – Brazil; 9 Brazil Universidade Federal de Uberlândia, Uberlândia, MG – Brazil; 10 Brazil Universidade Estadual do Rio de Janeiro, Rio de Janeiro, RJ – Brazil; 11 Brazil Instituto do Coração, InCor, São Paulo, SP – Brazil; 12 USA American Heart Association Inc, Dallas, Texas – USA; 13 USA University of North Carolina at Chapel Hill, Chapel Hill, North Carolina – USA

**Keywords:** Cardiovascular Diseases/complications, Pandemic, COVID-19, Heart Failure, Public Hospitals, Mortality, Delivery of Health Care/methods

## Introduction

Brazil is one of the most hit countries by the pandemic caused by the novel coronavirus (Coronavirus Disease, COVID-19), with more than half million deaths in June, 2021, the world’s second-highest number of deaths.^1^ It has been reported that the COVID-19 outbreak has affected the delivery of care of patients without COVID-19 (non-COVID-19 patients), particularly patients with cardiovascular (CV) diseases, increasing the death toll from the pandemic. Studies have shown a reduction in hospitalizations in tandem with an increase mortality from CV diseases during the outbreak in many countries.^2 - 8^ However, most of these reports focused on patients with acute coronary syndrome (ACS ) in developed countries, with few data on heart failure (HF) admissions, and the reasons for the worse mortality are not well understood. We evaluated the changes in severity, reasons for decompensation and 30-day mortality of patients admitted for HF in public hospitals included in the Best Practices in Cardiology (BPC) program in Brazil during the COVID-19 outbreak and compared with previous periods.

## Methods

We performed a cohort study using data from the BPC program, which is an ongoing program adapted from the Get With The Guidelines initiative, to improve quality of cardiovascular care and patients’ outcomes in Brazil. The study design, rationale and procedures have been previously published.^9^ We included consecutive patients aged 18 years or older admitted with decompensated HF in tertiary public hospitals affiliated to the Brazilian Unified Health System (SUS) during the COVID-19 outbreak from March 12^th^, 2020 to October 31^st^, 2020 (epidemiological weeks 11 to 44) and compared them with patients admitted in the same hospitals during the same epidemiological weeks in 2019 and 2018.

Baseline HF-specific characteristics were prospectively collected during hospitalization, by trained local investigators, using medical charts and structured interviews with the patient. The Acute Decompensated Heart Failure National Registry (ADHERE) algorithm score was calculated using a stepwise approach according to systolic blood pressure, creatinine and urea nitrogen blood levels to stratify HF patients into low, intermediate and high risk.^10 , 11^ The outcome was all-cause death in a 30-day follow up. Death and date of death were ascertained through medical records, next of kin and death certificates.

Informed consent was obtained from every patient and this study was approved by the Coordinating Center’s Institutional Review Board (# 48561715.5.1001.0060).

### Statistical analysis

Continuous variables were evaluated for the normal distribution using the shape, skewness, and kurtosis of distribution, and Kolmogorov-Smirnov test if needed. We compared clinical characteristics, measures of severity and treatment between study periods using the unpaired t test for normally distributed variables, which were presented as mean ± standard deviation, and the Mann-Whitney U test for non-normally distributed variables, presented as median (25th percentile, 75th percentile). Categorical data was presented as proportions and compared using the chi-square test. To assess the association between study periods and the outcomes, we calculated Kaplan-Meier estimates and compared the probability of all-cause mortality using the log-rank test. A Cox (proportional hazards) regression analysis adjusted for age, sex, etiology of HF (ischemic, Chagas, other), left ventricular ejection fraction (LVEF), cardiac resynchronization therapy (CRT), implantable cardioverter defibrillator (ICD), and education levels, and stratified by income levels, chronic kidney disease (CKD) and previous HF was performed; the proportional hazard assumption was tested based on Schoenfeld residuals, with no violation for the proportional hazard assumption for the studied groups. P-values<0.05 were considered statistically significant. Analyses were performed using Stata version 15.1 (Stata Corp., College Station, TX).

## Results

During the epidemiological weeks 11 to 44 in the years of 2018, 2019 and 2020, 1,084 patients were admitted for HF in seven eligible centers, representing six states ( *Alagoas, Bahia, Ceará, Minas Gerais, Pará* and *São Paulo* ) in Brazil. From these, we excluded 218 patients due to missing systolic blood pressure (n=86), creatinine (n=43), blood urea levels (n=20) or loss to follow-up (n=69). The final analysis was performed in 866 patients admitted for HF.

Compared with the previous two years, we observed a 20% reduction in the number of patients admitted for HF during the COVID-19 outbreak, particularly in the first four months (9.2 ± 4.2 vs 11.6 ± 3.0 admissions per week during *vs* . before outbreak, respectively).

During COVID-19 outbreak, most baseline characteristics remained the same, but LVEF was lower than before the outbreak. Ischemic heart disease and Chagas disease were the most common etiology of HF, and history of CKD was more prevalent during the pandemic. Also, severity of HF at admission seemed to be worse during COVID-19 outbreak. Although clinical profiles did not differ between study periods, patients admitted during COVID-19 outbreak had significantly worse ADHERE risk score ( Table 1 ).

**Table 1:** Characteristics at admission of patients hospitalized for acute heart failure before and during COVID-19 outbreak

	Before COVID-19 Outbreak (2018 to 2019)	During COVID-19 Outbreak (2020)	p value

	n=637	n=229	
Age, years	59.83 ± 16.00	61.00 ± 14.05	0.33
Women, n(%)	275 (43.2)	93 (40.6)	0.50
BMI, Kg/m^2^*	26.16 ± 5.56	27.03 ± 6.30	0.05
Etiology*			**0.028**
Ischemic	109 (17.1)	47 (20.5)	
Chagas	54 (8.5)	31 (13.5)	
Other	474 (74.4)	151 (65.9)	
LVEF, %	43.51 ± 17.62	37.33 ± 15.07	< 0.001
Previous HF, n(%)	395 (62.0)	153 (66.8)	0.20
CKD, n(%)	66 (10.4)	50 (21.8)	< 0.001
CRT, n(%)	3 (0.5)	3 (1.3)	0.19
ICD, n(%)	44 (6.9)	16 (7.0)	0.97
Low education, n(%)	265 (41.6)	119 (52.0)	0.007
Low income, n(%)*	469 (73.7)	170 (74.6)	0.81
Length of stay, days	19.0 [10.0. 33.0]	17.0 [9.0. 28.0]	0.17
**Clinical profile**			**0.62**
Warm-dry	56 (10.3)	13 (7.7)	
Warm-wet	368 (67.9)	114 (67.9)	
Cold-wet	92 (17.0)	34 (20.2)	
Cold-dry	26 (4.8)	7 (4.2)	
**ADHERE risk, n(%)**			**0.009**
Low	290 (45.5)	79 (34.5)	
Intermediate	319 (50.1)	134 (58.5)	
High	28 (4.4)	16 (7.0)	

BMI: body mass index; HF: heart failure; CKD: chronic kidney disease; CRT: cardiac resynchronization therapy; ICD: implantable cardioverter defibrillator; BUN: blood urea nitrogen. *Clinical profile was missing for 156 patients; BMI was missing for 56 patients; income level was missing for 2 patients; LVEF was missing for 23 patients.

All-cause mortality of patients hospitalized for HF significantly increased during the COVID-19 outbreak ( Figure 1 ). In the 30-day follow-up period, 50/637 (7.8%) and 31/229 (13.5%) patients died before and during the outbreak, respectively. After adjusting for potential baseline confounders, the risk of death in 30 days was approximately two times higher in patients admitted during the COVID-19 outbreak (adjusted HR = 1.89 [95% CI 1.19, 3.03]; *p* =0.007), as compared with the two previous years.

**Figure 1:** Kaplan-Meier estimates of the probability of death in patients admitted for heart failure before and during the COVID-19 outbreak.

Before COVID-19 outbreak: epidemiological weeks 11 to 44 in 2018 and 2019; during COVID-19 outbreak period: epidemiological weeks 11 to 44 in 2020

Poor compliance to either dietary and medication treatment recommendations, and acute kidney disease were more commonly reported as factors triggering HF decompensation during the COVID-19 outbreak, as compared with the previous period ( Figure 2 ). Infection as reason for decompensation during the COVID-19 outbreak was not different as compared with before the outbreak.

**Figure 2:** Factors triggering HF decompensation according to study period;.ACS: acute coronary syndrome; AKD: acute kidney disease.

* p<0.05 from Chi-square tests comparing the proportions between the two periods; before COVID-19 outbreak: epidemiological weeks 11 to 44 in 2018 and 2019; during COVID-19 outbreak period: epidemiological weeks 11 to 44 in 2020

## Discussion

In this large registry of HF hospitalizations in Brazil, our main findings were: 1) the 30-day mortality increased roughly twice during the COVID-19 outbreak compared with before the pandemic; 2) patients with HF were more likely to decompensate due to poor treatment compliance and acute kidney disease; and 3) they were admitted in worse condition, as indicated by a worse ADHERE risk score. These findings help understand the impact of the pandemic in patients with HF in Brazil, affecting patients’ behavior, disrupting the delivery of healthcare, and increasing the risk of dying from acute HF.

So far, there are few reports of the impact of the COVID-19 pandemic on HF patients. Similar to our findings, studies from Germany and England found that the short-term mortality significantly increased among patients admitted for HF during the beginning of the COVID pandemic, but this was not found in later studies from Denmark.^2 , 4 , 5^ This indicates that patients with CV diseases have been differently affected depending on the country-level response to the pandemic. Our study showed an increase in mortality from acute HF in a largely affected country, and that this impact has lasted longer than the first few months of the outbreak.

The reasons for the higher mortality are multifactorial. Non-adherence to dietary and drug treatment as factors triggering HF decompensation during the COVID-19 pandemic may provide insights on the mechanisms for these worse outcomes. Treatment of HF is complex, and it often requires a patient-centered multidisciplinary approach to improve long-term treatment compliance.^12^ The worse outcomes from acute HF may reflect a disruption in the delivery of care in the outpatient setting. Ambulatory services were interrupted, and cardiology teams were rearranged and transitioned to the care of COVID-19 in many centers, shifting the focus from effective measures that are essential to reduce mortality of this population. Moreover, loss of social support due to social isolation may have hampered continuity of treatment, particularly among vulnerable individuals.

It should be noticed that guidance to manage CV diseases during the COVID-19 pandemic have mainly focused on ACS, but patients with HF have also been largely affected by the pandemic. Efforts should be made to continue providing adequate care for these patients.^13^ Healthcare policies targeted to this population, including strategies to maintain delivery of outpatient care, such as telemedicine and remote monitoring, may help reduce mortality during the pandemic.

Our study has limitations: we included only public hospitals, most of them university hospitals. Also, all hospitals in our study participated in the BPC program, which aims to improve quality of cardiovascular care and patients’ outcomes, and this may not reflect HF admissions in all public hospitals in Brazil. Finally, our results do not represent those patients who were not admitted or who did not to come to the hospital.

## Conclusion

In this large registry of patients admitted for HF in public hospitals included in the BPC program in Brazil, one of the most-affected countries by the COVID-19 pandemic, poor treatment compliance and acute kidney disease were the most common reasons for HF decompensation, and the 30-day mortality increased twice during the COVID-19 outbreak, as compared with previous periods. Public health strategies in response to the pandemic should ensure maintenance of care to HF patients, particularly in the most affected countries.

## * Supplemental Materials

For additional information, please click here.
